# Hand Hygiene Knowledge and Practices among Domestic Hajj Pilgrims: Implications for Future Mass Gatherings Amidst COVID-19

**DOI:** 10.3390/tropicalmed5040160

**Published:** 2020-10-16

**Authors:** Hashim Mahdi, Amani Alqahtani, Osamah Barasheed, Amjad Alemam, Mohammed Alhakami, Ibrahim Gadah, Hadeel Alkediwi, Khadijah Alzahrani, Lujain Fatani, Lamis Dahlawi, Saeed Alsharif, Ramon Shaban, Robert Booy, Harunor Rashid

**Affiliations:** 1 National Centre for Immunisation Research and Surveillance, The Children’s Hospital at Westmead, Westmead, NSW 2145, Australia; robert.booy@health.nsw.gov.au (R.B.); harunor.rashid@health.nsw.gov.au (H.R.); 2 Discipline of Child and Adolescent Health, Faculty of Medicine and Health, The University of Sydney, Sydney, NSW 2006, Australia; 3 College of Health Sciences, Saudi Electronic University, Jeddah 23442, Saudi Arabia; 4 Saudi Food and Drug Authority (SFDA), Riyadh 13312, Saudi Arabia; amani.shelwa@gmail.com; 5 The Executive Administration of Research and Innovation, King Abdullah Medical City in Holy Capital (KAMC-HC), Makkah 24246, Saudi Arabia; barasheed.o@kamc.med.sa; 6 Faculty of Dentistry, Umm Al-Qura University, Makkah 24381, Saudi Arabia; amjadalemam@gmail.com; 7 College of Medicine, King Saud Bin Abdulaziz University for Health Science, Jeddah 22384, Saudi Arabia; muhammedfuzan21@gmail.com; 8 College of Applied Medical Sciences, King Saud Bin Abdulaziz University for Health Science, Jeddah 22384, Saudi Arabia; ot.ibraheem@gmail.com; 9 College of Pharmacy, Umm Al-Qura University, Makkah 24381, Saudi Arabia; hadeel.alkediwi@hotmail.com (H.A.); khadijah.a.z17@gmail.com (K.A.); 10 College of Nursing, Umm Al-Qura University, Makkah 24381, Saudi Arabia; lujainM.fatani@gmail.com; 11 Ghaya Community Pharmacy, Makkah 24234, Saudi Arabia; lamisdah0@gmail.com; 12 Command and Control Centre of Infectious Diseases of Public Health Department of Ministry of Health, Taif 26521, Saudi Arabia; dr.saeedalsharif@gmail.com; 13 Faculty of Medicine and Health, Susan Wakil School of Nursing and Midwifery & Marie Bashir Institute for Infectious Diseases and Biosecurity, The University of Sydney, Sydney, NSW 2006, Australia; ramon.shaban@sydney.edu.au; 14 Department of Infection Prevention and Control, Division of Infectious Diseases and Sexual Health, Westmead Hospital and Western Sydney Local Health District, Westmead, NSW 2151, Australia

**Keywords:** COVID-19, Hajj, hand hygiene, infection prevention and control, infectious disease, mass gathering

## Abstract

This study examined Hajj pilgrims’ knowledge and reported practice of hand hygiene. In Hajj 2019, a cross-sectional survey was undertaken in Mina, Makkah, Saudi Arabia, of domestic Saudi pilgrims aged ≥18 years by using a self-administered Arabic questionnaire that captured data on pilgrims’ socio-demographics, hand hygiene knowledge, and reported practices of hand cleaning following certain actions. A total of 348 respondents aged 18 to 63 (median 32) years completed the survey, of whom 200 (57.5%) were female. The mean (±standard deviation (SD)) hand hygiene knowledge score was 6.7 (±SD 1.9). Two hundred and seventy one (77.9%) and 286 (82.2%) of respondents correctly identified that hand hygiene can prevent respiratory and gastrointestinal infections respectively, but 146 (42%) were not aware that it prevents hand-foot-mouth disease. Eighty-eight (25.3%) respondents erroneously reported that hand hygiene prevents HIV. Washing hands with water and soap was the most preferred method practiced before a meal (67.5% (235/348)), after a meal (80.2% (279/348)), after toilet action (81.6% (284/348)), when hands were visibly soiled (86.2% (300/348)), and after waste disposal (61.5% (214/348)). Hajj pilgrims demonstrated a good knowledge and practice of hand hygiene, but there are gaps that are vital to control outbreaks such as COVID-19.

## 1. Introduction

COVID-19 pandemic has affecting about 38 million individuals with over one million deaths around the world (as of 13 October 2020) [1]. The disease is believed to transmit from human to human primarily via respiratory droplets, direct contact and indirect contact. Most mass gatherings (MGs), therefore, have been cancelled during this pandemic. MGs are defined as the concentration of people at a specific location for a specific purpose over a set period of time and which has the potential to strain the planning and response resources of the country or community [2]. MGs are known to accelerate the progression of a pandemic. A MG of two million people in Mexico, the Iztapalapa Passion Play that took place in 5–11 April 2009 is believed to have accelerated the spread of the last pandemic caused by influenza A(H1N1)pdm09 [3]. Each year, Hajj pilgrimage to Makkah, in Saudi Arabia, attracts two to three million people from around the world making it one of the largest annual MGs. Respiratory tract infections are the leading illnesses during Hajj affecting 40–90% of pilgrims [4]. Such religious and other MGs pose a significant risk for the spread of infectious diseases, as currently the case with the global pandemic of COVID-19. Saudi Arabia has reported about 338 confirmed cases with about 5000 fatalities [1], and despite banning of Umrah (minor pilgrimage to holy cities in Saudi Arabia) and subsequently downscaling the Hajj of 2020, there have been clusters of COVID-19 in Makkah and Medina, the two important pilgrimage cities [5].

MG-related confirmed cases of COVID-19 have been reported in South East Asian countries like Malaysia [6], and South Korea [7]. In the Middle East, the virus spread from a religious MG in Qom, a Shi’ite holy city (120 km south of the capital Tehran), Iran [8]. Intense crowding during Hajj is highly likely to amplify the risk of transmission of COVID-19. Approximately a third of Hajj attendees are elderly or have pre-existing health conditions, rendering them highly vulnerable to severe form of disease and fatality from infection [9].

In the absence of a definitive preventive measure, non-pharmaceutical measures such as social distancing, isolation, quarantine, and hand hygiene remain the mainstay of infection prevention and control against respiratory infections including COVID-19 [10].

The Saudi Arabian Ministry of Health (MoH) recommends an array of preventive measures annually to minimize the risk of transmission of respiratory infections during Hajj season, including hand washing, respiratory hygiene, and vaccinations [9]. Despite this advice, research demonstrates that the uptake of preventive measures varies among pilgrims [11].

Hand hygiene was found to be the most favored infection preventive measure for Hajj attendees [11]. It is significantly associated with a reduction in self-reported respiratory infections including influenza-like illnesses (ILI) [12], frequency of sputum production, myalgia [13], and fever [14]. Use of an alcohol-based hand sanitizer significantly reduces *Streptococcus pneumoniae* detection in respiratory samples [15]. There is a paucity of focused research that has examined knowledge and practice of hand hygiene among domestic Hajj attendees. This study examined hand hygiene knowledge and reported behaviors among Saudi Hajj pilgrims in 2019 and attempts to inform policy for the other MGs amidst COVID-19.

## 2. Materials and Methods

A cross-sectional study using a paper-based anonymous survey was conducted among Saudi pilgrims aged ≥18 years who attended Hajj in 2019 in Mina tent city, Greater Makkah, Saudi Arabia, a place where pilgrims spend at least four nights during Hajj. A convenient sampling strategy was used to recruit participants. The data collectors went to Mina and randomly approached the nearest two domestic tour groups (Hamlahs) who agreed to cooperate with the study. From these selected Hamlahs the sample was drawn by randomly asking pilgrims to participate in the study.

This paper-based self-administered anonymous survey was conducted in Arabic. Initially, a survey questionnaire in English was drafted using some exemplary questions used in published surveys [16,17]. Two public health researchers and one epidemiologist translated the questionnaire to Arabic independently. Subsequently, two professional translators performed backward translation separately (who have not seen the original survey or material). The survey underwent pilot testing via a focus group of eight respondents to discuss and answer the survey to ensure readability, personal interpretation of individual question and solving any problems in answering the questions. Following revision, the final version was prepared by the authors that addressed grammatical discrepancies.

The questionnaire consisted of three parts. Part 1 collected non-identifying participant sociodemographic data. Part 2 collected data about the participant’s knowledge of hand hygiene using true/false questions. Additionally, there were questions on common myths and fallacies of hand hygiene reported in the literature, such as the misconceptions that hands should be held under water while lathering with soap and the adequate time used for hands rubbing before rinsing. This was assessed using a Likert scale that uses scoring from 0 to 12, with a higher score indicating considerable knowledge level on hand hygiene and a lower score indicating insufficient knowledge. Part 3 comprised of items related to self-reported hand washing behavior. Respondents were asked whether they washed their hands, and by what methods, in different situations during their Hajj journey. These included hand washing before and after eating, after using toilet, after caring a sick person, when hands are visibly dirty, after disposal of a garbage bag, after sneezing or coughing, and after handshaking.

Seven senior medical students (four males and three females) were selected as volunteers and trained as data collectors. The volunteers approached and explained the study purpose and methodology to domestic Saudi Hajj pilgrims residing in camps in Mina on 11th and 12th of August 2019. Pilgrims who agreed to participate were then given the questionnaires to complete, and respondents’ queries, if any, were answered. This being an anonymous survey, no signed consent was obtained, and respondents’ completion of the survey was considered as their implied consent. Ethics approval was obtained from King Abdullah Medical City, Makkah, Saudi Arabia (IRB ref 19-558).

To ensure correct and uniform entry of data, the collected data from hard copy questionnaires were entered into an electronic form using the Google Forms software (Google LLC, Mountain View, CA, USA) (https://forms.gle/syQt5ouVzJ5usr318). Subsequently, all the data were exported to a master Excel spreadsheet (Microsoft Office 356, version 2002, Redmond, WA, USA) for cleaning and coding before importing to Statistical Package for Social Sciences (SPSS) software (IBM SPSS Statistics for Windows, version 25.0, IBM Corp, Armonk, NY, USA). Descriptive statistics for socio-demographic characteristics, hand hygiene knowledge level, and hand hygiene practices of the respondents were reported. Where appropriate the difference between categorical variables was examined using the chi-squared test, and independent t-test was used to compare the gender differences in the knowledge score on hand hygiene. Binary logistic regression, using the backward Wald method, controlling for factors, such as age, gender, chronic medical conditions, educational level, employment status and the number of times respondents attended Hajj, was used to investigate variables related to hand hygiene knowledge and practices. Previous researchers found that at least 60% of respondents were practicing hand hygiene at Hajj; considering an error margin of 5% to be acceptable for this anonymous survey, a sample of 370 respondents was considered sufficient for this study.

## 3. Results

Volunteers approached and invited 380 pilgrims to participate in the study, and 348 (91.6%) agreed. The median age of respondents was 32 (range 18–63) years, 200 (57.5%) were female. Of all 348 respondents, 208 (59.8%) had a bachelor’s degree or above, 108 (51.7%) were employed, and 43 (12.4%) reported having at least one chronic disease. Just over three-quarters (270, 77.6%) of the respondents reported attending Hajj that year for the first time, with the rest (78, 22.4%) reporting attending Hajj previously at least once (Table 1).

Of the 348 respondents, there were 230 (66.3%) who reported being aware of the annual Saudi MoH health recommendations for Hajj travelers issued, 262 (75.5%) who reported seeking some form of health advice before Hajj journey, 286 (82.2%) reported receiving the compulsory meningococcal vaccine, and 288 (82.8%) reported receiving other recommended vaccines (Table 2).

With respect to knowledge about hand hygiene, the mean (±standard deviation (SD)) of total scores (0 to 12) was 6.7 (±1.9). Less than half (155, 44.5%) of the respondents had a low knowledge score (defined as total score of ≤6), over half (175, 50.3%) had a medium score (defined as total score of between 7 and 9), and the rest (18, 5.2%) had a high score (defined as total score of ≥10). In multivariate logistic regression analysis, there was no significant association between the level of hand hygiene knowledge and possible factors, including gender, age, having chronic diseases, number of Hajj times, education, and employment status (all *p* values > 0.05). Nonetheless, both gender and employment status showed a near statistical significance (odds ratio (OR) = 1.73, 95% CI = 0.98–3.07, *p* = 0.06 and OR = 1.76, 95% CI = 0.97–3.16, *p* = 0.06, respectively) with females and employed respondents having higher knowledge level compared to males and unemployed individuals. The results of the respondents’ level of hand hygiene are detailed in Table 3.

Table 4 summarizes the results of whether pilgrims practiced hand hygiene or not and by what methods in different situations during Hajj. Except for cleaning hands following handshakes, which was reported by just over half (177, 51.9%) respondents, an overwhelming majority of respondents reported cleaning their hands following other tasks. Hand washing using water and soap was the most commonly reported hand hygiene method among pilgrims across all situations. A small number (58, 16.7%) of the respondents reported barriers to using hand hygiene during Hajj season, notably unavailability of soap and hand rub (60.5% (31/58)), limited access to washrooms (23.3% (18/58)) and intense crowding (16.3% (14/58)).

## 4. Discussion

This study explored hand hygiene knowledge and reported practices of domestic Saudi Hajj pilgrims. There is a paucity of research that has examined hand hygiene knowledge among Hajj pilgrims. In this study the pilgrims had a moderate knowledge of hand hygiene (6.7 ± 1.9). Most respondents had an accurate knowledge of the protective role of hand hygiene against common infectious diseases such as respiratory and gastrointestinal infections, but some were not aware of its role against some less common but nonetheless important infections. Concerningly, about 40% of respondents were not aware that hand hygiene can prevent hand-foot-mouth disease for which hand hygiene is strictly advised, and a quarter of respondents mistakenly thought hand hygiene prevented HIV infection. Pilgrims’ knowledge also varied for responses pertaining to questions relating to common myths surrounding hand hygiene and knowledge on correct hand hygiene procedure. For instance, half of the respondents reported that persistent hand washing lowers immunity, half thought that temperature of the water makes a difference in terms of the cleansing effect of hand washing, a third did not know that hands should not be placed under water while lathering, about 58% erroneously thought rubbing hands just for 10 s is enough to ensure disinfection while it should be 20 s, and about a third thought 40% alcohol is sufficient for disinfecting hands whereas the correct answer should be minimum 60% alcohol [10].

A study conducted among Malaysian pilgrims during the 2018 Hajj showed 87.1% pilgrims knew that washing one’s hands with hand sanitizers can protect one from flu-like illness, which compares with only 77.9% pilgrims in our study knowing the role of hand hygiene against flu/cough/ILI [18]. The lower proportion in our study could be explained by the fact that foreign pilgrims are generally better informed and better prepared for Hajj travel since health authorities in their countries of origin are mandated to ensure health advice to pilgrims on communicable diseases prompting months of preparation including attendance at pre-travel health seminar (usually more than once) before embarking on Hajj journey [9]. It is also encouraging to see that more domestic pilgrims sought pre-Hajj advice in the present study compared to the previous year (three-quarters versus half) [19]. In this study, there was no significant difference in knowledge level by gender or age as was found in other studies involving Saudi healthcare workers and trainees [20]. It is unsurprising that some pilgrims lacked knowledge in some more specialist themes like alcohol concentration needs to be in disinfectants, duration of time required for hand rubbing, prohibition of placing hands under water while lathering and insignificance of water temperature, yet the pilgrims seem to have similar or even better knowledge in some domains compared to community dwellers in another developed country in Asia [17].

Hand washing with soap and water was the most common type of hand cleaning reportedly used by the study respondents in almost all the situations followed by alcohol hand rubbing. The results of this study are consistent with, and corroborate, the findings of other studies, including a systematic review, which found among Hajj pilgrims hand washing with soap was more popular than hand gel or sanitizers [18,21]. In contrast, a study explored domestic pilgrims’ uptake of health preventive measures during the peak Hajj days found the proportion of respondents washing their hands with soap and cleaning hands with hand sanitizers was same (65%); however, pilgrims who were concerned about food poisoning were more likely to clean their hands with hand sanitizers (adjusted OR 2.5, 95% CI 1.1–5.4) indicating pilgrims’ degree of concern may dictate the mode of hand hygiene [19].

The results of the present study also showed a relatively poor hand hygiene behavior after touching a patient with only 39.7% washing hands with soap–water and 31.9% using alcoholic hand rub, after sneezing and coughing (respectively 25.6% and 19.5%) and following handshakes (respectively 19.5% and 18.1%). Similarly, low compliance with hand hygiene following these actions in previous studies, for instance 15% Australian pilgrims washed hands after touching a patient while at Hajj, despite the fact that 86% made an intention before Hajj to wash hands after touching an ill person, indicating there were practical issues that barred them [22], e.g., unavailability of soap and hand rub, limited access to washrooms and intense crowding reported as barriers to hand hygiene in this study. Another study conducted among members of public across Gulf countries showed that only 39% individuals washed their hands with soap after handshakes which may indicate that in a non-epidemic setting most people would not wash hands following handshake [23]. Regardless of what products are being used by pilgrims, hand hygiene in general has been proven to be an effective measure against respiratory infections at Hajj [12,13,14] and is practiced by pilgrims from different nationalities [18,19].

The patchy knowledge gap and non-compliance on certain occasions warrant improvement. This is indicated by the findings from this and a previous study that showed pilgrims with a university-level education had a higher hand hygiene compliance compared to those with a lower education, and several other studies reporting lack of awareness as an important hindrance to hand hygiene, meaning education may improve the hand hygiene uptake [24]. Through a pre- and post-intervention survey conducted during the Hajj 2011, Turkestani and colleagues showed that direct health education to pilgrims is effective in improving hand hygiene compliance rate from 79.1% to 95.5%. [25]. During a pandemic era, such as the current COVID-19 outburst, intensive pre-travel health education perhaps through a certification program following a short course on hygiene may be made compulsory for all pilgrims. Tour operators may conduct the course and should have, as a pre-requisite of running Hajj tours, more advanced knowledge of hygiene, health, and safety. This can be buttressed by direct health education and a random quick knowledge test at the points of entries supplemented by multi- lingual health messages on billboard with graphical illustration on how to perform hand hygiene, map a direction of hand hygiene facilities and resources. Effective pandemic health messaging during the Hajj 2009 was associated with higher compliance with protective measures and with shorter duration of respiratory illnesses [12]. Furthermore, giving advice by Islamic scholars about the importance of alcohol- based hand rubs use and reinforce how this practice does not harm pilgrims could potentially eliminate taboos surrounding the use of alcohol-based hygienic products and in turn enhance compliance [26].

Although a small number of respondents reported some barriers in practicing hand hygiene during Hajj such as unavailability of soap and hand rub, limited access to washrooms, and intense crowding, solutions, for example, providing supplementary hand hygiene products to pilgrims, could potentially increase the uptake. However, in Hajj setting, such improvements are not always feasible; therefore, pilgrims are encouraged to carry their own personal hygiene products.

Considering the continuance of the COVID-19 pandemic and its risks of spreading in mass gatherings, and the increase in average infections globally, the Saudi Government decided to downscale Hajj for this year 2020 allowing only a thousand local and resident foreigners perform the pilgrimage [27].

This study had some limitations. Firstly, the survey was conducted only among domestic Saudi pilgrims who unlike international pilgrims bypass many vicissitudes of travel hence these findings may not be generalizable. Secondly, the reported barriers were not qualitatively gauzed, and finally, being anecdotal in nature some data (e.g., vaccination history) could be at risk of recall bias. These limitations can be addressed by a mixed-method study involving an extended sample from multiple nationalities with different health, education and cultural backgrounds.

## 5. Conclusions

There is a paucity of research that has examined infection prevention and control measures in MGs. Hand hygiene was generally acceptable among domestic Saudi pilgrims but there were variable knowledge gaps in some aspects that may be improved by intensive health education and awareness-raising strategies. Hajj pilgrims demonstrated a good knowledge and reported practice of hand hygiene, although there were some gaps in their key areas that are vital to containing and mitigating outbreaks, particularly in the context of MGs and the current global COVID-19 pandemic.

## Figures and Tables

**Table 1:** Demographic characteristics of respondents (N = 348).

Variables	n (%)
*Age, years*	
Median (range)	32 (18–63)
*Gender*	
Male: Female	148:200 (42.5:57.5)
*Education*	
None	11 (3.2)
Primary/elementary school certificate	22 (6.3)
High school certificate	73 (21)
Diploma	34 (9.8)
University degree	182 (52.4)
Higher degree	26 (7.5)
*Employment stats*	
Unemployed	126 (36.3)
Student	42 (12)
Employed	180 (51.7)
Self-employed	19 (5.5)
Full time worker	148 (42.5)
Casual/part-time worker	13 (3.7)
*Number of times attending Hajj*	
Once	270 (77.6)
More than one	78 (22.4)

**Table 2:** Awareness of Saudi Arabian Ministry of Health (MoH) recommendations, pre-Hajj health advice, and vaccination status (N = 348).

Variables	n (%)
*Awareness of MoH recommendations*	
No	117 (33.6)
Yes	231 (66.4)
*Sought pre-Hajj health advice*	
No	85 (24.4)
Yes	263 (75.6)
*Sources of Hajj health advice*	
Doctors	48 (11.1)
Special Hajj websites (e.g., MoH)	81 (18.8)
Tour groups	80 (18.5)
Family and fiends	130 (30.1)
General websites	93 (21.5)
*Vaccination status*	
Mandatory meningococcal vaccine	286 (82.2)
Recommended influenza vaccine	280 (80.5)

**Table 3:** Hand hygiene knowledge level among respondents (N = 348).

Questions	Answered n (%)
**Which of the following diseases can be transmitted by poor hand hygiene?**	
*Diarrhoeal diseases*	
True ^§^	286 (82.2)
False	62 (17.8)
*Flu, cough (upper respiratory infection or ILI)*	
True ^§^	271 (77.9)
False	77 (22.1)
*Hand-foot-mouth disease*	
True ^§^	202 (58)
False	146 (42)
*HIV/AIDS*	
True	88 (25.3)
False ^§^	260 (74.7)
*Skin infections*	
True ^§^	195 (56)
False	153 (44)
*Eye infections*	
True ^§^	258 (74.1)
False	90 (25.9)
*Diabetes*	
True	16 (4.6)
False ^§^	332 (95.4)
**Are the following statements correct?**	
*Always keeping your hands clean may lower our body defence mechanism*	
True	181 (52)
False ^§^	167 (48)
*Hands should be held under water while lathering with soap*	
True	120 (34.5)
False ^§^	228 (65.5)
*An alcohol-based hand sanitiser that contains 40% alcohol is sufficient for hands disinfectant*	
True	119 (34.2)
False ^§^	229 (65.8)
*Rubbing my hands until soap forms a lather for 10 s before rinsing is enough for hand disinfection*	
True	202 (58)
False ^§^	146 (42)
*Temperature of water makes no difference in terms of the cleansing effect of hand cleaning*	
True ^§^	147 (42.2)
False	201 (57.8)
**Mean (±SD) of Total Scores (0 to 12)**	6.7 (±1.92)

**^§^** Correct answer.

**Table 4:** Respondents’ hand hygiene practices and methods used in different situations during Hajj (N = 348).

Types of Hand Hygiene	Before Meal n (%)	After Meal n (%)	After Toilet Action n (%)	After Touch A Sick Person n (%)	When Hands Visibly Dirty n (%)	After Waste Disposal n (%)	After Sneeze/Cough n (%)	After Handshakes n (%)
Water only	60 (17.2)	29 (8.3)	33 (9.5)	33 (9.5)	12 (3.4)	44 (12.6)	60 (17.2)	30 (8.6)
Water and soap	235 (67.5)	279 (80.2)	284 (81.6)	138 (39.7)	300 (86.2)	214 (61.5)	89 (25.6)	68 (19.5)
Alcohol hand rubs	22 (6.3)	19 (5.5)	23 (6.6)	111 (31.9)	31 (8.9)	44 (12.6)	68 (19.5)	63 (18.1)
Water wipes	16 (4.6)	14 (4.0)	5 (1.4)	14 (4.0)	3 (0.9)	12 (3.4)	51 (14.7)	16 (4.6)
Did not practice hand hygiene	15 (4.3)	7 (2.0)	3 (0.9)	52 (14.9)	2 (0.6)	34 (9.8)	80 (23.0)	171 (49.1)

## References

[1] World Health Organization WHO Coronavirus Disease (COVID-19) Dashboard. https://covid19.who.int.

[2] World Health Organization Public Health for Mass Gatherings: Key Considerations. https://www.who.int/publications/i/item/public-health-for-mass-gatherings-key-considerations.

[3] Zepeda-Lopez H.M., Perea-Araujo L., Miliar-Garcia A., Dominguez-Lopez A., Xoconostle-Cazarez B., Lara-Padilla E., Ramirez Hernandez J.A., Sevilla-Reyes E., Orozco M.E., Ahued-Ortega A. (2010). Inside the outbreak of the 2009 influenza A (H1N1)v virus in Mexico. PLoS ONE.

[4] Petersen E., Memish Z.A., Zumla A., Al Maani A. (2020). Transmission of respiratory tract infections at mass gathering events. Curr. Opin. Pulm. Med..

[5] Ebrahim S.H., Memish Z.A. (2020). Saudi Arabia’s drastic measures to curb the COVID-19 outbreak: Temporary suspension of the Umrah pilgrimage. J. Travel Med..

[6] Che Mat N.F., Edinur H.A., Abdul Razab M.K.A., Safuan S. (2020). A single mass gathering resulted in massive transmission of COVID-19 infections in Malaysia with further international spread. J. Travel Med..

[7] Shim E., Tariq A., Choi W., Lee Y., Chowell G. (2020). Transmission potential and severity of COVID-19 in South Korea. Int. J. Infect. Dis..

[8] Ebrahim S.H., Memish Z.A. (2020). COVID-19-the role of mass gatherings. Travel Med. Infect. Dis..

[9] Algarni H., Memish Z.A., Assiri A.M. (2016). Health conditions for travellers to Saudi Arabia for the pilgrimage to Mecca (Hajj)-2015. J. Epidemiol. Glob. Health.

[10] World Health Organization (WHO) Coronavirus Disease (COVID-19) Advice for the Public. https://www.who.int/emergencies/diseases/novel-coronavirus-2019/advice-for-public.

[11] Benkouiten S., Brouqui P., Gautret P. (2014). Non-pharmaceutical interventions for the prevention of respiratory tract infections during Hajj pilgrimage. Travel Med. Infect. Dis..

[12] Balaban V., Stauffer W.M., Hammad A., Afgarshe M., Abd-Alla M., Ahmed Q., Memish Z.A., Saba J., Harton E., Palumbo G. (2012). Protective practices and respiratory illness among US travelers to the 2009 Hajj. J. Travel Med..

[13] Gautret P., Vu Hai V., Sani S., Doutchi M., Parola P., Brouqui P. (2011). Protective measures against acute respiratory symptoms in French pilgrims participating in the Hajj of 2009. J. Travel Med..

[14] Hashim S., Ayub Z.N., Mohamed Z., Hasan H., Harun A., Ismail N., Rahman Z.A., Suraiya S., Naing N.N., Aziz A.A. (2016). The prevalence and preventive measures of the respiratory illness among Malaysian pilgrims in 2013 Hajj season. J. Travel Med..

[15] Benkouiten S., Charrel R., Belhouchat K., Drali T., Nougairede A., Salez N., Memish Z.A., Al Masri M., Fournier P.E., Raoult D. (2014). Respiratory viruses and bacteria among pilgrims during the 2013 Hajj. Emerg. Infect. Dis..

[16] Suen L.K.P., So Z.Y.Y., Yeung S.K.W., Lo K.Y.K., Lam S.C. (2019). Epidemiological investigation on hand hygiene knowledge and behaviour: A cross-sectional study on gender disparity. BMC Public Health.

[17] Pang J., Chua S.W., Hsu L. (2015). Current knowledge, attitude and behaviour of hand and food hygiene in a developed residential community of Singapore: A cross-sectional survey. BMC Public Health.

[18] Dauda Goni M., Hasan H., Naing N.N., Wan-Arfah N., Zeiny Deris Z., Nor Arifin W., Abubakar Baaba A. (2019). Assessment of Knowledge, Attitude and Practice towards Prevention of Respiratory Tract Infections among Hajj and Umrah Pilgrims from Malaysia in 2018. Int. J. Environ. Res. Public Health.

[19] Alqahtani A.S., Althimiri N.A., BinDhim N.F. (2019). Saudi Hajj pilgrims’ preparation and uptake of health preventive measures during Hajj 2017. J. Infect. Public Health.

[20] Ra’awji B.A.A., Almogbel E.S., Alharbi L.A., Alotaibi A.K., Al-Qazlan F.A., Saquib J. (2018). Knowledge, attitudes, and practices of health-care workers regarding hand hygiene guidelines in Al-Qassim, Saudi Arabia: A multicenter study. Int. J. Health Sci. (Qassim).

[21] Alqahtani A.S., Fakeerh M., Bondagji D., Park S., Heywood A.E., Wiley K.E., Booy R., Rashid H. (2020). Hand hygiene compliance and effectiveness against respiratory infections among Hajj pilgrims: A systematic review. Infect. Disord. Drug Targets.

[22] Alqahtani A.S., Wiley K.E., Mushta S.M., Yamazaki K., BinDhim N.F., Heywood A.E., Booy R., Rashid H. (2016). Association between Australian Hajj Pilgrims’ awareness of MERS-CoV, and their compliance with preventive measures and exposure to camels. J. Travel Med..

[23] Alqahtani A.S., Rashid H., Basyouni M.H., Alhawassi T.M., BinDhim N.F. (2017). Public response to MERS-CoV in the Middle East: iPhone survey in six countries. J. Infect. Public Health.

[24] Alqahtani A.S., Wiley K.E., Tashani M., Willaby H.W., Heywood A.E., BinDhim N.F., Booy R., Rashid H. (2016). Exploring barriers to and facilitators of preventive measures against infectious diseases among Australian Hajj pilgrims: Cross-sectional studies before and after Hajj. Int. J. Infect. Dis..

[25] Turkestani A., Balahmar M., Ibrahem A., Moqbel E., Memish Z.A. (2013). Using health educators to improve knowledge of healthy behaviour among Hajj 1432 (2011) pilgrims. East. Mediterr. Health J..

[26] Wai Khuan N., Shaban R.Z., van de Mortel T. (2018). The influence of religious and cultural beliefs on hand hygiene behaviour in the United Arab Emirates. Infect. Dis. Health.

[27] Perveen S., Orfali R., Azam M.S.U., Aati H.Y., Bukhari K., Bukhari S.I., Al-Taweel A. (2020). Coronavirus nCOVID-19: A pandemic disease and the Saudi precautions. Saudi Pharm. J..

